# Consumer health risk assessment of Arsenic and Mercury in hen eggs through Monte Carlo simulations

**DOI:** 10.1186/s12889-023-16223-4

**Published:** 2023-07-10

**Authors:** Abdol-samad Abedi, Hedayat Hoseini, Fatemeh Mohammadi-Nasrabadi, Negar Rostami, Fatemeh Esfarjani

**Affiliations:** 1 Food and Nutrition Policy and Planning Research Department, Faculty of Nutrition Sciences and Food Technology, National Nutrition and Food Technology Research Institute (NNFTRI), Shahid Beheshti University of Medical Sciences, Tehran, Iran; 2 Department of Food Science and Technology, Faculty of Nutrition Sciences and Food Technology, National Nutrition and Food Technology Research Institute, Shahid Beheshti University of Medical Sciences, Tehran, Iran

**Keywords:** Hen eggs, Arsenic, Mercury, Risk assessment, ICP-MS, THQ, Monte Carlo simulations

## Abstract

**Background:**

This study was conducted to assess the concentration of heavy metals (arsenic and mercury) and estimate the probability that consumption of hen egg products collected in Iran has carcinogenic or non-carcinogenic consequences.

**Methods:**

A total of eighty-four hen eggs from 21 major brands were randomly selected from among thirty local supermarkets in two seasons (winter (January) and summer (August) 2022). Arsenic (As) and Mercury (Hg) was determined by using ICP-MS. The human health risk assessment refers to the formulation of the USEPA standard focused on Estimated Daily Intake (EDI), International Lifetime Cancer Risk (ILCR), Target Hazard Quotient (THQ), and Monte Carlo simulation (MCS) as a probabilistic method. Data analysis was carried out using the statistical software SPSS. Differences in mean concentrations of As and Hg in two seasons were tested by paired t-test.

**Results:**

Over two seasons, the average As and Hg concentrations in hen eggs were 0.79 and 0.18 µg.kg^−1^, respectively. Seasonal difference in As concentration (p = 0.451) was not significant, whereas that of Hg concentration (*p* < 0.001) was significant. The calculated value of EDI was 0.29 µg As/day and 0.06 µg Hg/day. The EWI in the maximum scenario of as level in hen eggs was estimated to be 8.71 µg As and 1.89 µg Hg/month for Iranian adults. THQ's mean for As and Hg in adults was determined to be 0.00385 and 0.00066, respectively. In addition, ILCRs by MCS for As were 4.35E-4.

**Conclusion:**

In total, the result indicates that there was not a significant risk of developing cancer; the calculation of THQ was still below the accepted level of 1, indicating that there was no risk while, according to most regulatory programs (ILCR > 10^− 4^) shows a threshold carcinogenic risk of arsenic through consuming in hen eggs. Therefore, policymakers need to be aware that it is prohibited to establish chicken farms in heavily polluted urban areas. It is essential to regularly conduct examinations to measure the presence of heavy metals in both ground waters used for agriculture and the feed provided to chickens. Additionally, it is advisable to focus on raising public awareness about the importance of maintaining a healthy diet.

## Background

The perfect balance and diversity in its high-quality nutrients along with its high availability and play a key role in the daily diet of individuals worldwide, its affordable price makes the conventional hen egg a food product [1–3]. However, eggs may contain a high concentration of heavy metals that come primarily from food and water feed, mainly impacted by the environment [4]. Environmental contamination brought on by the growth of livestock and poultry production has sparked worries about food safety, particularly about potential heavy metal residues in feed additives or poultry feed and products, including eggs [5]. Heavy metals can be transferred from poultry to eggs [1]. Consequently, hens can take up heavy metals from various environmental sources and pass those chemicals on to their eggs [6]. Season, location, chicken age, nutritional behaviors, and metabolic cycle are some of the elements that influence how much the laying hens absorb heavy metals [7].

Heavy metals, including arsenic (As) and mercury (Hg), can be hazardous and not biologically necessary even in small amounts [8, 9]. Inorganic As is a naturally occurring element found in the earth's crust and is known as the "king of poisons" due to its ability to induce liver and lung cancer [2, 10]. It is widely dispersed throughout the environment in the air, water, and land [10]. As is the substance that has raised the most significant questions regarding potential harmful effects on human health because it is easily transported across food chains, and is not known to perform any crucial biological functions. It has been demonstrated that children are more sensitive than adults, and the effects are cumulative [2]. Symptoms of As toxicity include abdominal pain, nausea, and diarrhea, which may lead to severe diseases including neurological, respiratory, reproductive, hepatic, and cardiovascular, as well as various cancers [11].

Mercury is one of the most hazardous metals after lead which has no known benefits for human physiology. It is still widely employed in the industry. An adult of normal weight, weighing 70 kg, is thought to have 13 mg of mercury in his body. In humans, high Hg levels are found in the skin, nails, hair, and kidneys. It is a highly reactive molecule that produces toxic effects by binding highly to sulfhydryl and a lesser degree to hydroxyl, carboxyl, and phosphoryl groups [12]. Mercury deposited in soil may continue to be discharged into surface waters for thousands of years because soils have a long residence time for the metal. Feed ingredients, such as the usage of fish and crops tainted with Hg, may also contaminate poultry feed while it is being processed. Plants can accumulate mercury from the atmosphere [13].

Additionally, heavy metals can increase oxidative stress by generating free radicals, which harms antioxidant defense. Long-term exposure to As and Hg can have harmful consequences even in small doses. The food chain can become contaminated as a result of the poisoning of the soil, water, plants, and animals by these harmful metals. As a result, exposure to heavy metals through inhalation, ingestion, skin contact, and drinking water can be hazardous to human health [14–18].

In many countries, including Iran, is an increasing prevalence of various types of cancer, and one of the main parameters affecting cancer is the environment. Microbial, chemical, and radiation contamination can affect the majority of cancer [19]. The contamination of the environment can also be detected in the food chain. The major sources of high metal contamination in the environment include mining, industry, household trash, pesticides, and agricultural practices [20].

Knowledge of the mineral content of eggs is becoming increasingly important for many reasons that are related to their health and nutritional value of eggs including, the consequences of egg metals on its embryonic development, and the use of eggs as bio-indicators for environmental metal pollution [21]. Tehran is one of the world's most polluted cities [22]. This pollution can affect human health directly or indirectly through contamination of the food chain. Chickens play an essential role in the food chain and their contamination can have irreparable consequences. Hen eggs may become tainted with heavy metals through chicken feed and drinking water, both of which are primarily influenced by the environment. Consistently consuming heavy metals in food at hazardous levels may have negative impacts on humans by impairing a variety of biological and metabolic systems [19].

Iran, the Middle East’s largest poultry producer, produces up to 1.2 million tons of eggs annually [23]. Policymakers and risk managers, in particular, can receive comprehensive information through risk assessments [24]. It is possible to identify complicated cause-and-effect linkages and reduce risk using even modest estimates of risk variance. The environmental health risk assessment technique is so naturally cautious and strongly protective of human health [25].

There have been limited studies published about the levels of As and Hg in hen eggs. There have been limited studies published about the levels of As and Hg in hen eggs, Iran faces problems with poultry feed because of the sanctions, and hen eggs are one of the most popular foods in the food baskets of the majority of low-income households. This study was also conducted under the framework of a heavy metal monitoring plan and risk assessment in the food chain, according to the results of which policymakers can design plans for how to reduce these heavy metals and improve guidelines for protecting consumer health and increasing food safety.

So this study for the first time evaluated consumer health risk assessment of arsenic and mercury in hen eggs through Monte Carlo simulations.

## Data and methods

### Samples

In one week in two seasons (winter (January) and summer (August) 2022), the top twenty-one egg brands in Tehran were sampled from among thirty local supermarkets in five districts (North, Sought, Center, West, and East) of Tehran. Forty-two samples were randomly selected from 21 brands (two eggs chosen from each batch content of 12 eggs) for each season. A total of 84 fresh, unbroken, and unfertilized hen eggs were selected after being visually assessed by candling. They were then cleaned with deionized water, coded, and packed in polyethylene zip bags. Refrigerated conditions were used to transport items to the laboratory for chemical analysis. Samples were quickly chilled at 4 °C before being prepared. Instead of using metal tools, chemically stable sterile flacon tube tools were utilized to prevent any chemical contamination. The analytical preparation was carried out immediately. To avoid contamination elements before use, the entire piece of equipment was cleaned with diluted HNO3 (10%) and then distilled water [26].

### Samples preparation

Carefully, the egg's contents were separated from the eggshell. The next stage involved mixing and homogenizing each whole egg (yolk and white) component before pouring it into Petri dishes to be dried in an oven for 24 h at 70 °C to become a fine powder.

To digest 0.5 g of dried egg samples, 10 ml of 70% nitric acid and 30% hydrogen peroxide (v/v) were purchased from Merk (Darmstadt, Germany) and left at room temperature for one night.

The digestion took place for 4 h at 150 °C until the solution was clear. The solution was cooled to room temperature (22–23 °C), diluted with deiodine water to 50 ml, and filtered through 0.45 L acid-resistant filter paper. The solution was stored at 4 °C for later analysis. The liquefied solution was filtrated and diluted with 20% HNO3 before being analyzed by ICP-MS (ULTIMA2, 6100 DRC-e Perkin Elmer Elan). In addition, the glassware containers used for analysis were first washed with detergent and rinsed several times with tap water several times and then they have soaked overnight in 6 N HNO3 (Merk) solutions and finally rinsed with deionized water.

According to the FDA Elemental Analysis Manual, samples were assessed for total As and Hg using heat-block-assisted acid digestion and the ICP-MS technique. The blank solution was made in the same way but without an egg. For quality assurance, blanks and certified standards were analyzed after every ten samples. The samples were analyzed in triplicates. Inductively coupled plasma mass spectrometry (ICP-MS) is a type of mass spectrometry that uses an inductively coupled plasma to ionize the sample. It is known and used for its ability to determine metals and several non-metals in liquid samples at very low concentrations (ppb = parts per billion = µg/l). It is well-recognized that this technique is the fastest and most reliable for determining the content of heavy metals in the food business [26].

The goal of a quality assurance and quality control (QA/QC) program is to monitor the quality of data from sampling in the field through the generation of the final results, to ensure that both the user and external parties are confident in the quality of the data obtained. Furthermore, a properly designed and implemented QA/QC program will also identify errors and the potential stage of the analysis. To ensure proper quality, a QA/QC program is therefore aimed at understanding the following [27, 28]:

### Sampling:

- At an appropriate location from five districts in Tehran and in two seasons,
- Taking the sample properly,
- Storing the sample not too long
- The sampling equipment was plastic and stainless steel
- Double-checking sample labels before starting each test

### In laboratory:

- Regularly calibrating equipment and machines.
- Consistently and regularly documenting testing methods.
- Procuring personal and lab-wide certifications.
- Sterilizing equipment and preventing personal contamination.
- Regularly evaluating standard procedures carried out by lab techs and interns.
- Running both a triple sample and a blank sample to compare test results.

All the mentioned above have been tried to be included as (QA/QC) in the present study and also all parameters for ICP-Mass were used as shown in the Table 1.

**Table 1:** Conditions of ICP-MS apparatus for determining Arsenic and Mercury in hen eggs

Parameter	value
Radiofrequency	1200W(40 MHz)
Plasma gas (Argon) flow	16 l/min
Nebulizer gas (Argon) flow	1 l/min
Read delay and analysis speeding	30 s
Wash	60 s
Wash speeding	30 rpm
Dwell time	50 ms
Resulting/amu10%peak	0.7
Integration time	3.5
Linear working (total element) ppb	0.053
Precision%RSD ( n = 10)	1.3
Addition/Recovery	93–103
Repetition	3
LOD (As, Hg)	0.00033 μg kg^−1^
LOQ (As, Hg)	0.001 μg kg^−1^

### Health risk assessment

In this study, the assessment of human health risk is used to describe the potential risk of heavy metals from consuming hen eggs obtained from Iranian commercial hen eggs. The United State Environmental Protection Agency (US EPA) proposed the method to calculate health risk needs (estimated daily intake, target hazard quotient, and carcinogenic risks) [29].

The average body weight of adult consumers, the mean amounts of these metals in eggs, and the number of eggs ingested were used to determine the Estimate daily intake (EDI) for Hg and As.

The EDI of As and Hg was calculated according to Eq. 1.

1 $$\mathrm{EDI} = \mathrm{FIR} \times \mathrm{CM} / \mathrm{WAB}$$

EDI is the estimated daily intake (µg kg ^−1^ b.w/day); F_IR_, is the consumption of the daily eggs (ml/day-1); C_M_, is the mean level of metal (mg /mL^−1^); and W_AB_, is the average body weight (kg). According to the National Institute of Nutritional Research and Food Industry of Iran research [30], adults (18 to 50) in Iran consume 25.4 g of eggs daily. Also, the W_AB_ (body weight) for adults is 70, according to body weight studies by the United States Environmental Protection Agency (EPA) [31, 32]. As and Hg exposure from eating eggs was calculated. In addition to EDI, estimated weekly intake (EWI, g kg -1 b.w/week) and estimated monthly intake (EMI, g kg -1 b.w/month) of As and Hg for adults were computed to compare with the PTWI and PTMI, respectively, set by JECFA (Joint FAO/WHO Expert Committee on Food Additives) [31]. The PTWI of As and Hg was established at 15 and 4(µg kg^−1^ BW/week) by JECFA [33].

### Non-carcinogenic risk estimation

Target Hazard Quotient (THQ) estimation was performed to determine the non-carcinogenic risk among hen egg consumers [34, 35] by Eq. 2.

2 $$\begin{array}{c}{Daily\;intake}\hspace{0.17em}=\hspace{0.17em}{Conc\;metal\;in\;egg\;}({mg}/{kg})\hspace{0.17em}\times \hspace{0.17em}{Average\;per\;capita\;consumption\;of\;egg\;}({mg}/{day})\\ \mathrm{THQ}=\frac{\mathrm{EF }\times \mathrm{ ED }\times \mathrm{ FIR }\times \mathrm{ CM }}{\mathrm{RFD }\times \mathrm{ WAB }\times \mathrm{ TA}}\times {10}^{-3}\end{array}$$

According to research, 365 days per year and 70 years were determined to be the frequency of exposure (EF) and the exposure time equivalent to the mean lifetime in Iran. As and Hg had oral reference doses (RFD) of 0.0003 and 0.0001(mg kg^−1^ BW/day), respectively [36]. TA (exposure duration for non-carcinogens) was 25,550 days.

### Carcinogenic risk estimation

To assess the potential cancer risk of As in people who consume eggs [36], the Incremental Lifetime Cancer Risk (ILCR) was computed from Eq. 3.

3 $$\mathrm{ILCR} = \mathrm{EDI} \times \mathrm{CSF}$$

A lifetime means the dosage of 1 mg kg-1 BW/day results in a risk known as the cancer slope factor (CSF) [19] 1.5 mg kg-1 day of CSF for As [36].

According to the U.S. Environmental Protection Agency (US-EPA), the safe limit for cancer risk is below approximately 1 chance in 1,000,000 lifetime exposure (ILCR < 10^–6^), the threshold risk limit (ILCR > 10^–4^) for a chance of cancer is above 1 in 10,000 exposure, where corrective measures are significant, and the moderate risk level (ILCR > 10^–3^) is above 1 in 1,000 where public health safety assessment is more critical [37, 38].

### Statistical analysis

After gathering the necessary data, data analysis was done using the SPSS software (SPSS Inc., version 16, Chicago, IL, USA). The frequency (%), mean (Standard Error), and median (minimum–maximum) for both normal and non-normal distributions were used to summarize data for categorical variables. The Kolmogorov–Smirnov test was used to determine whether the data were normal. A paired t-test was used to determine significant seasonal differences between paired egg samples. Statistics were considered significant for *P*-values under 0.05.

The calculation of the limit of detection, LOD (µgkg^−1^)_**,**_ was based on.the measurement results obtained with blank filters in the present study for As and Hg was 0.0003. The results were given in micrograms per kilogram of the sample's moist weight. The limit of quantification (LOQ) was estimated to be 0.001 for both metals [39].

### Monte Carlo simulation (MCS) technique

There may be some uncertainties in the estimate of health risks [36, 40]. When single-point data are utilized to estimate health hazards caused by exposure to pollutants such as toxic metals, a high level of uncertainty is seen. To lessen the uncertainty in the assessment of health risks, MCS was used in our experiment as a probabilistic method [41, 42]. For the creation of risk assessment models, Oracle, Inc.'s Crystal Ball software (version 11.1.2.4, USA) was utilized. A percentile of 95% of THQ and ILCR in the cumulative probability graph is the threshold for threatened exposed populations in this study, which included 10,000 repeats [43].

## Results and discussion

Eggs are a great source of protein and other vital elements. In many commercial and homemade dishes, it is also a common ingredient. Therefore, everyone must be aware of the trace-element content of eggs [44]. The number of eggs consumed rises daily. Consumption may vary depending on various variables, including socioeconomic position, age group, and urban versus rural residence. Compared to poorer people, who eat boiled or fried eggs, the higher classes consume more eggs in cakes, biscuits, and salads [44].

### Heavy metals hen egg residue of As and Hg in winter and summer

The results of this study showed that the mean residue of As were 0.52 and 01.07 μg kg^−1^ in the winter and summer, respectively (Table 2). In total, the average concentration of As was relatively low (0.79 μg kg^−1^), especially when compared with the data in other countries such as Italy 7 μg kg^−1^ [39], France 8 μg kg^−1^ [45], the United Kingdom 0. 9 μg kg^−1^ [15], Turkey 2.96 μg kg^−1^ [20], Bangladesh 30 μg kg^−1^ [46], and Belgium 16 μg kg^−1^ [47]. In two investigations conducted in Iran, the mean levels of As in hen eggs were determined to be 30 μg kg^−1^ [48] and 8 μg kg^−1^ [49], respectively.

**Table 2:** Comparison of As and Hg residue (µg kg^−1^) in hen eggs (two seasons)

**Heavy metals**	**Min**	**Max**	**Mean ± SE**
**AS**			
Winter	0.10	1.50	0.52 ± 0.050
Summer	0.00	7.50	1.07 ± 0.557
**Hg**^**a**^			
Winter	0.00	0.50	0.26 ± 0.021
Summer	0.10	0.10	0.10 ± 0.00

^a^Differences in As between winter and summer were not significant with Paired t-test (*p* = 0.451) but were significant in Hg (*p* < 0.001)

Moreover, based on the current study in Table 2, the average Hg levels were 0.26 and 0.10 μg kg^−1^ in winter and summer, respectively. The results showed the level of Hg in hen eggs was similar to or less than those of other countries (0.18 μg kg^−1^), such as China 0.1 μg kg^−1^ [50], France 4 μg kg^−1^ [45], the United Kingdom 1 μg kg^−1^ [15], Belgium 2 μg kg^−1^ [47], Turkey 0.34 μgkg^−1^ [20], and Denmark 2 μg kg^−1^ [51]. In a few studies conducted in Iran, the concentration of Hg was 70 μg kg^−1^ [49] and 26 μg kg^−1^ [4].

Overall, findings demonstrated that, compared to past Iranian assessments, As and Hg levels have decreased through these years. Differences in As concentration was not significant in the two seasons, according to the paired t-test result (*p* = 0.451); however, differences in Hg concentration (*p* < 0.001) were significant, which can be the sum of the following factors involved in the difference. The safety of poultry feed and any potential risks to human health have become significant concerns as commercial production of poultry and poultry feed has advanced on a bigger scale [52].

### Challenges and threats in heavy metal residues in hen eggs

Globally, pollution and technological growth are posing enormous challenges and threats to both humans and animals as follows:

1. Use of insecticides and poisonous plants on crops [53]
2. Antibiotics used in chicken feed, both therapeutic and non-therapeutic [54]
3. Lead, Mercury, Arsenic, Antimony, and other heavy metals have contaminated feed through water, crops, and industrial waste [52].

However, the primary source of poultry feeding and watering is based on mineral and agricultural products and water. There is still a chance that hazardous environmental elements or food additives could contaminate chicken feed, so this must be monitored appropriately. The presence of heavy metals in the chicken feed may be caused by several components, including minerals, additions for marine feed (such as fish meal, algae), trace elements (copper sulfate, zinc oxide), Roxarson (kills parasites and improves meat color), and anti-caking chemicals. As and Hg are a significant concern due to their poisonous qualities and the absence of a necessary biological function [55, 56].To the point that European Commission has set maximum limits on heavy metals, such as lead, cadmium, and mercury, in certain foods but not for hen eggs (European Commission Regulation, EC No 18812006) [57].

### Health risk assessment

In addition to assessing the total concentration of As and Hg by comparing the permissible limits, other factors, including exposure time, per capita intake, metal toxicity, and body weight, are crucial in assessing the possible health risk. Appropriate data interpretation was used to carry out exposure and non-carcinogenic and carcinogenic risk assessments for adults [19, 35].

### Exposure assessment

Dietary exposure of As and Hg through consuming hen eggs was assessed by calculating EDI, EWI, and EMI in two scenarios of the overall and maximum concentrations of these metals and compared to provisional tolerable weekly intake (PTWI) and provisional tolerable monthly intake (PTMI) established joint FAO/WHO Expert Committee on Food Additives( JECFA). The results were summarized in Table 3.

**Table 3:** EDI, EWI, EMI of As and Hg in adults due to consumption of hen eggs in two seasons

As	EDI^a^	EWI^b^	EMI^c^
**Winter**	0.19	1.32	5.66
**Summer**	0.39	2.72	11.65
**Mean**	0.29	2.03	8.71
**Hg**	EDI	EWI	EMI
**Winter**	0.09	0.66	2.83
**Summer**	0.03	0.25	1.09
**Mean**	0.06	0.42	1.89

^a^*EDI* Estimated Daily Intake, ^b^*EWI* Estimated Weekly Intake,^c^*EMI* Estimated Monthly Intake

The PTWI of As and Hg was established at 15 and 4 (µg kg^−1^ BW/week) by JECFA

The risk values (PTWI and PTMI) depend on the amount of consumption, the pollution rate of the desired food, and the weight of the target group. The PTWI of As and Hg was established at 15 and 4( µg kg^−1^ BW/week) by JECFA [33].

Therefore, the weekly intake of AS and Hg (EWI) from ingested eggs (in the maximum scenario) were 2.03 and 0.25 µg/week for an adult with 70 kg body weight, respectively (EDI: 0.29 As/day and 0.06 µg Hg/day for an adult) the EWI in the maximum scenario of as level in eggs were estimated to be 2.03 As and 0.42 Hg (µg/week) for Iranian adults in Table 3.

In 2010, the JECFA stated that daily ingestion of As through food consumption has minimal effect on overall exposure due to its long half-life. Therefore, tolerable and dietary ingestion of As for assessing the long-term and short-term health risks should be determined over 1 month or several months, respectively. Considering the maximum level of As and Hg in hen eggs, the EMI of adults was 8.71 and 1.89(µg/month), respectively.

In the present study, the exposure of As and Hg through consuming eggs (EWI and EMI) was lower than the risk values suggested, which indicates low risk for consumers [58]. It should be highlighted that this study only refers to the consumption of hen eggs, which may only contribute minimally to Iranian consumers' overall exposure to As and Hg.

### Non-carcinogenic risk

**Fig. 1:**
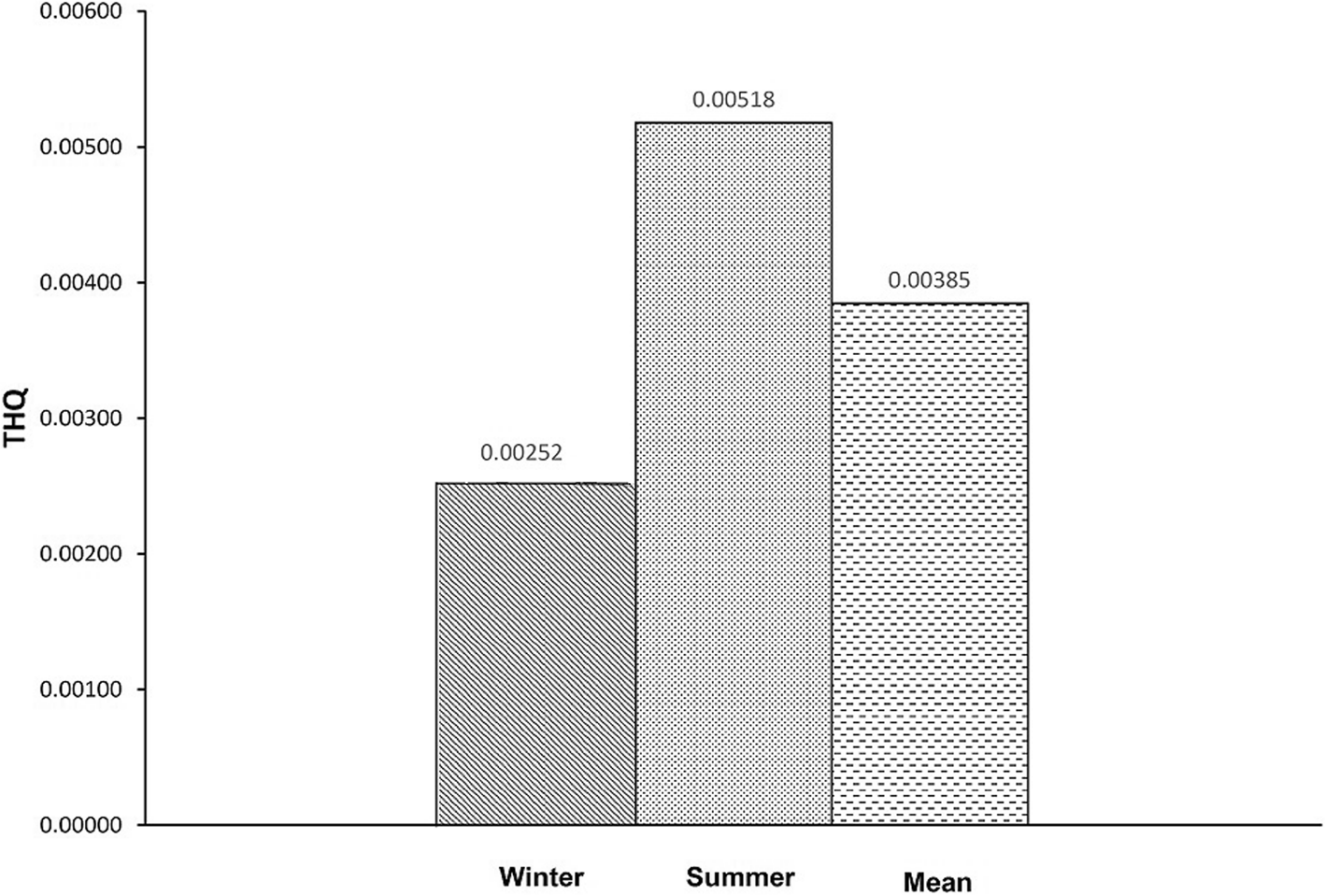
Target hazard quotient (THQ) of As in two seasons (winter, summer, and mean) through hen eggs consumption

**Fig. 2:**
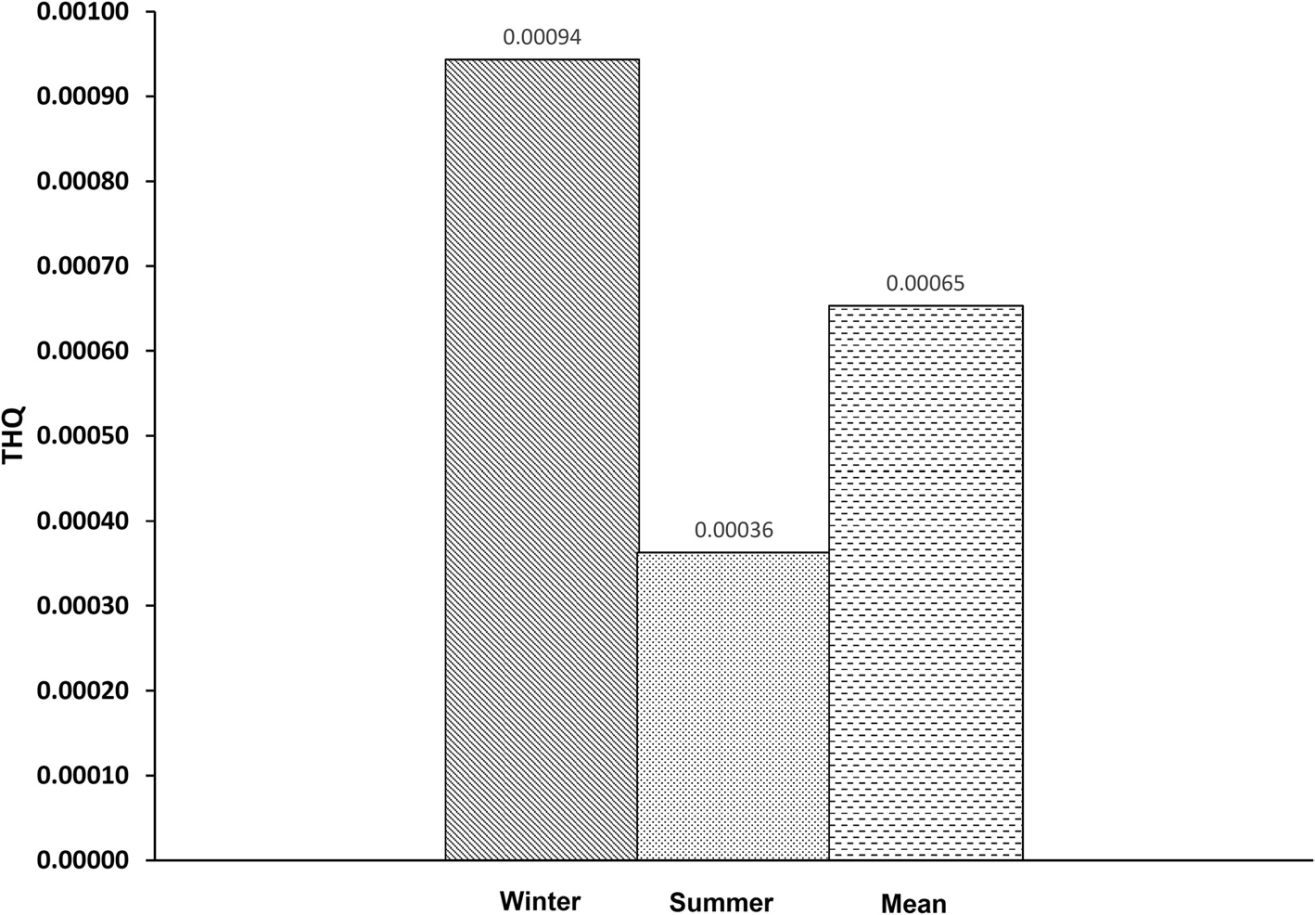
Target hazard quotient (THQ) of Hg in two seasons (winter, summer, and mean) through hen eggs consumption

The non-carcinogenic risk of As and Hg in consumers was calculated by determining the THQ (Target Hazard Quotient) value. The results of the ICP-MS analysis were utilized to calculate the adult population's overall and maximum levels of THQ for As and Hg’, as shown in Figs. 1 and 2. This value has been acknowledged as an appropriate variable for assessing the dangers associated with eating hazardous metals through contaminated food.

The THQ is described as the ratio of a contaminant's observed amount to the drug's reference oral dosage (RFD) [59]. Negative consequences may happen when a metal’s THQ value is more than 1, but they are less probable to occur when it is lower than 1 [60].

Based on the total amount of hen eggs consumed daily by Iranian adults (25.4 g/day) [30], the THQ’s mean of As and Hg for adults was calculated to be 0.00385 (winter 0.00252, summer 0.00518) and 0.00065 (winter 0.00094, summer 0.00036), respectively.

Several authors in other countries [61], including Bangladesh (0.260) (-), Belgium (3.539) (0.065), China (0.195) (-), Egypt (0.069) (-), Germany (3.006) (0.161), India (0.270) (-), Italy (0.072) (-), Malaysia (2.228) (-), USA (2.270) (8.511), and South Korea(-) (0.116) have reported adult As and Hg THQ values from eating hen eggs, respectively.

**Fig. 3:**
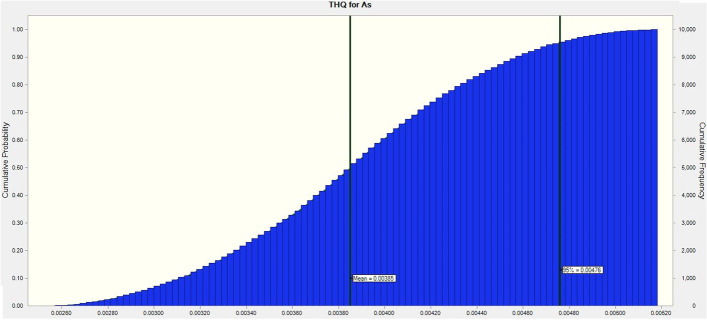
THQ distribution for As through Monte Carlo simulation

**Fig. 4:**
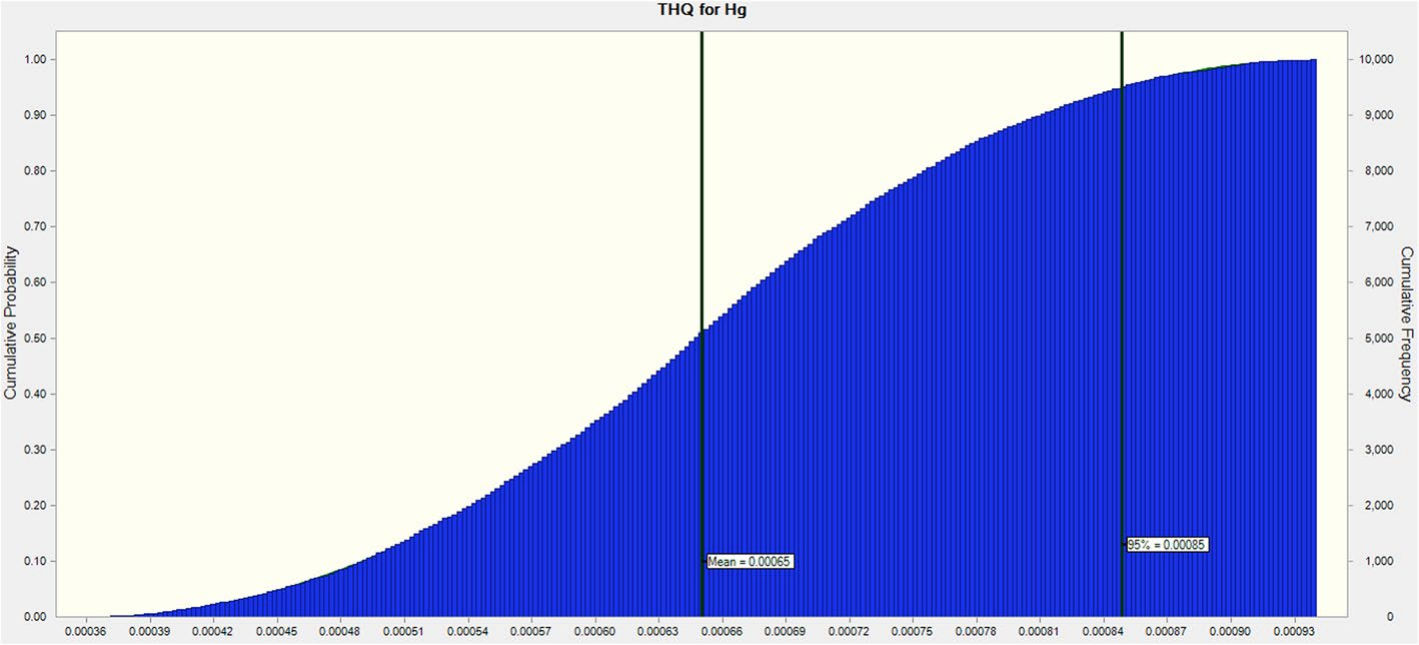
THQ distribution for Hg through Monte Carlo simulation

Because the THQ values of As and Hg for adult consumers in this research indicate values below one, the outcomes of this investigation demonstrate that eating eggs did not pose any risks to the health of Iranian consumers. In addition, the MCS revealed that the estimated THQ values for As and Hg for adults at the 95% percentile were 0.00385 and 0.00066, respectively, indicating that Iranian consumers are not possibly at risk for health issues as a result of consuming eggs (Figs. 3 and 4). It's essential to keep in mind that there are other ways to be exposed to these dangerous metals, including through water, skin contact, inhaling dust, and eating other foods. In this regard, a study conducted recently in Iran revealed that various heavy metal concentrations in tea samples were higher than the levels allowed by Iran National Standard and WHO [17]. A study conducted in Iran in 2022, proved that using depilatory products on the skin would not increase the risk of cancer or other serious illnesses, but continued usage might be harmful because of the excessive accumulation of these heavy metals [16]. As the most widely used tobacco in the world, cigarettes are also well-known. The assessment of the levels of heavy metals in smoked and non-smoked cigarettes in Iran revealed that heavy metals in cigarette butts can have both potentially harmful and beneficial consequences for the health of smokers who are subjected to inhalation [18].

This would indicate a severe risk to the health of the exposed population.

### Carcinogenic risk

Although a variety of characteristics, such as age, race, and gender, may contribute to the development of cancer, several studies have shown that exposure to environmental pollutants, such as toxic elements, increases the risk of cancer [62]. The ILCR value was applied to the current investigation's calculations to determine the carcinogenic risk for adult egg consumers. ILCR was only calculated for arsenic since the cancer slope factor (CSF) for Mercury's risk of oral cancer hasn't yet been determined.

There might be considerable ambiguity when estimating health risks [40]. When single-point data are utilized to evaluate health risks related to exposure to pollutants such as toxic metals, a high level of uncertainty is seen. As a consequence, MCS was used in our study as a probabilistic strategy to lower the uncertainties in the assessment of health risks.

**Fig. 5:**
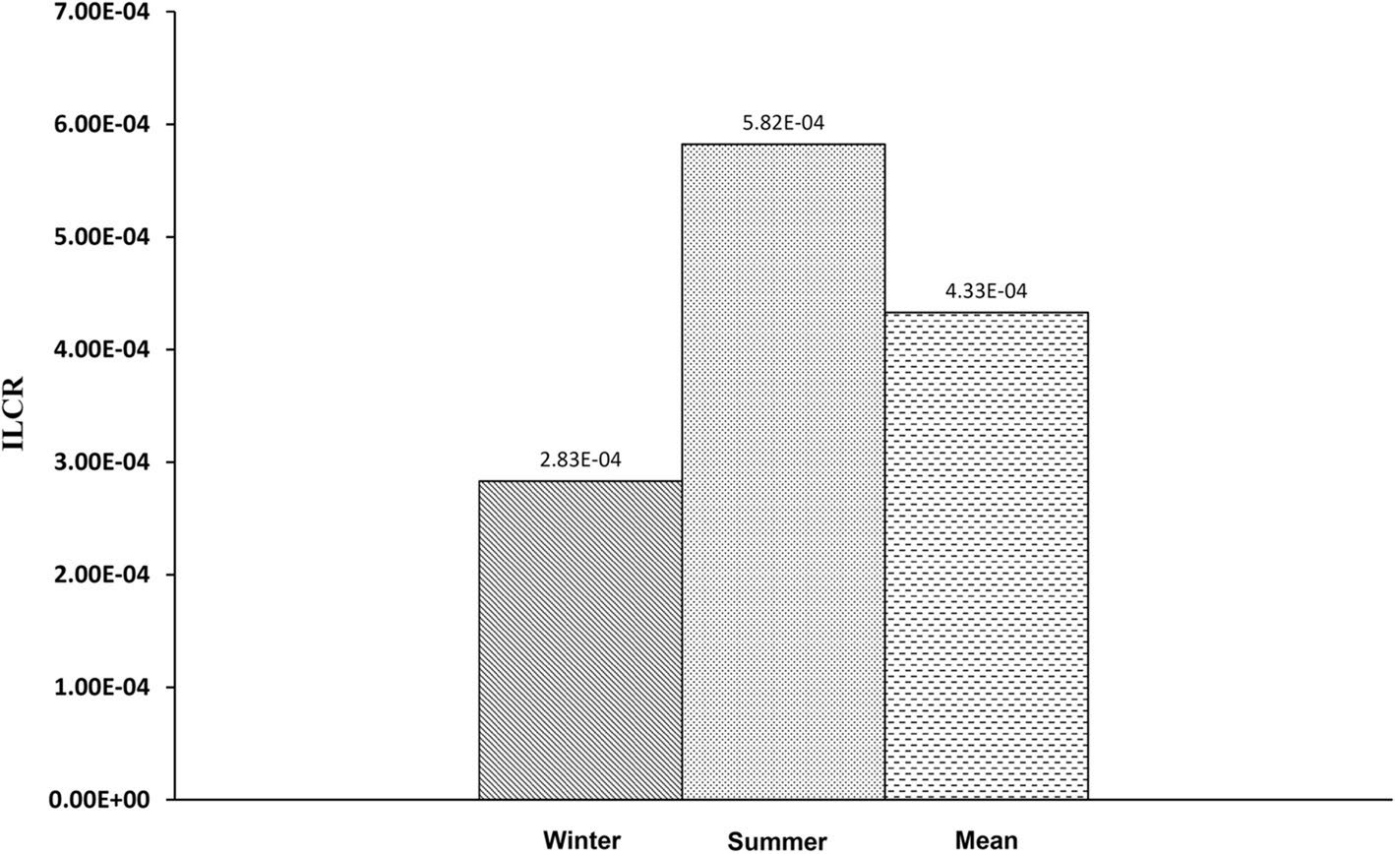
Incremental lifetime cancer risk (ILCR) of As in adults through hen eggs consumption in two seasons (winter, summer, and mean)

**Fig. 6:**
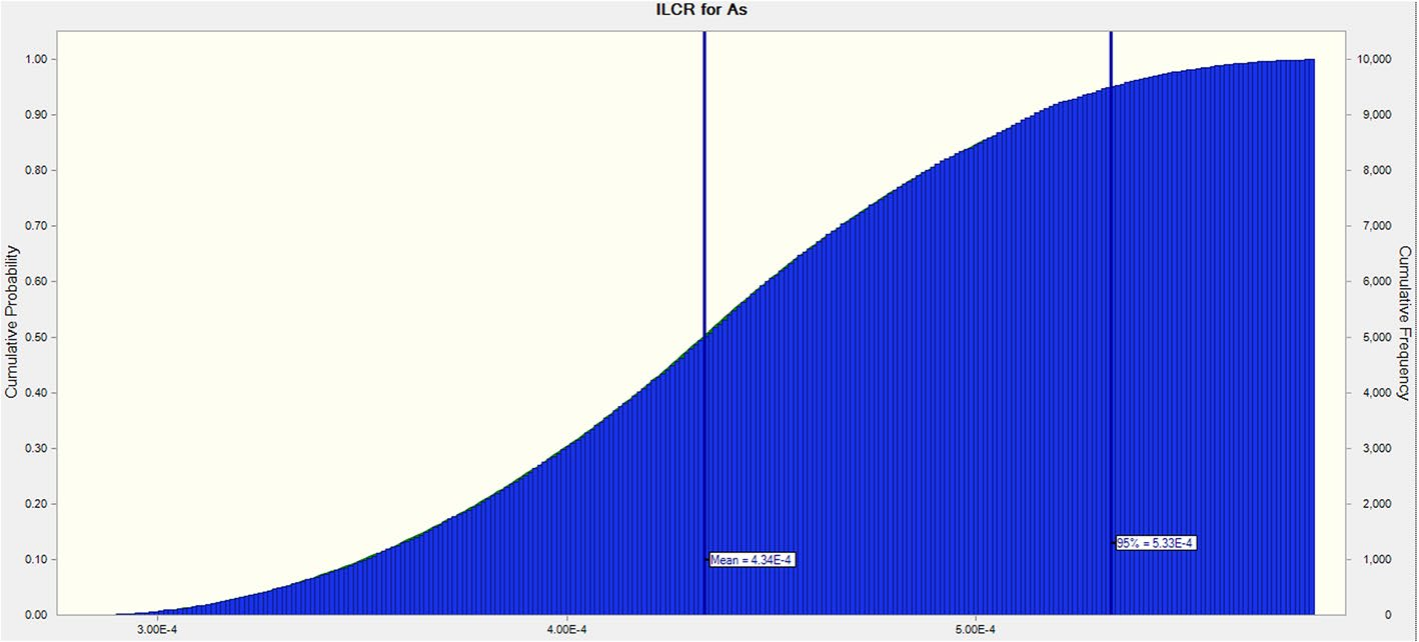
ILCR distribution for As through Monte Carlo simulation

It was calculated that the mean ILCR for As in adults was 4.33E-04 (winter: 2.83E-04, summer: 5.82E-04), which indicated the threshold risk limit (ILCR > 10^–4^) for a chance of cancer is above 1 in 10,000 exposure (Figs. 5 and 6).

These contaminants enter hen eggs through contaminated food, unclean water sources, and additional factors like age, species, and lying cycles. Other elements influencing the quality of hen eggs include supplemental nourishment and insecticides used to control pests [63]. According to most studies, contaminated feed consumed orally by hens can more frequently lead to the contamination of eggs [64].

Interestingly, our data described the amount of As and Hg in hen eggs as lower than in the majority of studies in other countries. The intake of staple foods such as rice raises major health problems due to the level of As and Hg in Iranian food. Further risk-based monitoring studies should be recommended to reduce exposure to As and Hg from other food sources. Human exposure to arsenic is a complex issue because it is closely related to the exposed population's environmental pollution, occupation, lifestyle, and dietary patterns. While drinking water has traditionally been considered a principal contributor to consumer exposure to arsenic, recent studies have shown that food represents an even more significant source of exposure to arsenic [65, 66]. Recently a study by Mohammad Pour, et al. (2023) showed by Monte Carlo simulation that water intake rate and mercury concentration were the most critical parameters in the hazard index for children and adults in Shiraz, Iran [67]. Environmental toxins like heavy metals can impact the safety and quality of hen eggs. Pesticides like phosphate fertilizers and contaminated air are the main sources of heavy metals in soil and crops. Heavy metals are absorbed from plant roots, moved to leaves, and stored in tissues depending on the kind and variety of plants, the type of sound water supply, the duration of irrigation, metal ionization, and transfer parameters [68]. Such as a study in Shiraz (Iran) (2023),heavy metals residue in fruit were As:7.5, Hg:4.38 µg.kg^−1^ [69].

Most research has shown that the concentration of As in groundwater in various geographic regions was beyond the limits set by WHO and the National Standards of Iran, another issue with heavy metals in groundwater resources [70]. The findings of an investigation conducted in Tehran, Iran, in 2015 indicated that the amount of heavy metals in the hen eggs collected was less than the permitted levels and therefore regarded as safe. The continual monitoring of these contaminants in the food chain was, however, strongly recommended by policymakers due to the significance of food contamination for public health [48, 49, 71].

Chemical contamination rates are greatly influenced by the distances that should be kept between drinking water supplies and industrial, mining, and agricultural activities. Farmers can reduce heavy metal pollution in crops, which results in the food that is hen eggs, by using fertilizer and implementing surveillance systems for agricultural areas. The national regulations for these contamination issues in hen eggs' food products have not established legally binding limitations.

Additionally, Particular attention has been made to exposure to children under the age of five because there are no reliable standard limits for As and Hg in hen eggs. In response to the requests of the food safety authorities, it is recommended that the international adopt this restriction. The presence of heavy metals in chicken feed, water, and meat should be assessed individually in future investigations.

### Limitation

Our samples might not represent all the hen egg samples in Iran. In addition, this study only looked at the heavy metals in hen eggs and not necessarily other food consumption. Thorough consideration of arsenic speciation among foods is necessary for reliable evaluations of exposure to inorganic arsenic in the food supply. It is possible to overestimate exposures that suggest a considerably higher risk than is present if arsenic exposure is expressed in terms of total arsenic and results are compared to the RFD for inorganic arsenic.

## Conclusion

Overall, findings demonstrated that, compared to past Iranian assessments, As and Hg levels have decreased through these years. Hen egg consumption by Iranian consumers did not pose a non-carcinogenic risk, based on THQ values of these hazardous metals below one. Moreover, the incremental lifetime cancer risk (ILCR) of As was estimated to be 4.33E-04, indicating that consumers in Iran are at the threshold carcinogenic risk of As through consuming hen eggs (ILCR > 10^–4^). However, exposure to As and Hg through food consumption, water consumption, skin contact, and inhalation can be harmful to human health. It is advised that As and Hg levels be frequently checked in hen eggs and other foods in Iran. In general, consumer consumption rates are connected with exposure to heavy metals through food chains, which may have a cancer-causing effect on people over time. Particular attention has been made to exposure to children under the age of five because there are no reliable standard limits for As and Hg in hen eggs. In response to the requests of the food safety authorities, it is recommended that the international adopt this restriction.

Therefore, policymakers need to be aware that it is prohibited to establish chicken farms in heavily polluted urban areas. It is essential to regularly conduct examinations to measure the presence of heavy metals in both groundwater used for agriculture and the feed provided to chickens. Additionally, it is advisable to focus on raising public awareness about the importance of maintaining a healthy diet.

## Data Availability

The datasets used and/or analyzed during the current study are available from the corresponding author upon reasonable request.
